# Subjective Beliefs About Trust and Reciprocity Activate an Expected Reward Signal in the Ventral Striatum

**DOI:** 10.3389/fnins.2019.00660

**Published:** 2019-06-26

**Authors:** Kim Fairley, Jana Vyrastekova, Utz Weitzel, Alan G. Sanfey

**Affiliations:** ^1^Institute of Tax Law and Economics, Department of Economics, Leiden University, Leiden, Netherlands; ^2^Donders Institute for Brain, Cognition and Behavior, Centre for Cognitive Neuroimaging, Radboud University, Nijmegen, Netherlands; ^3^Institute for Management Research, Radboud University, Nijmegen, Netherlands; ^4^Faculty of Law, Economics and Governance, Department of Economics, Utrecht University School of Economics, Utrecht, Netherlands; ^5^Behavioral Science Institute, Radboud University, Nijmegen, Netherlands

**Keywords:** trust, beliefs, reward, risk, uncertainty, ventral striatum

## Abstract

There is overwhelming evidence that the evaluation of both reward decisions and their associated outcomes are closely linked with bilateral activation of the ventral striatum, with these insights stemming from tasks such as the monetary incentive delay task for lotteries and multiround Trust Games for social settings. The essential element in these tasks is an externally provided cue associated with specific gains/trustworthy partners and losses/non-trustworthy partners. However, in reality people typically use their own beliefs to guide their decision-making and assess the likelihood of positive or and negative outcomes. As when participants assess the relationship between cues and rewards, individuals should anticipate rewards in correspondence to their beliefs, i.e., the higher the belief of obtaining a reward in the future, the higher the anticipation of reward. In this study, we use decision-makers’ own, naturally occurring, beliefs about both social and non-social contexts to examine the subsequent outcome of their choices. We hypothesize that mechanisms of belief-mediated reward processing are mediated by neural activation in the ventral striatum. An essential feature of our design is the elicitation of individuals’ beliefs prior to the decision-making task itself. Furthermore, our incentivized, non-deceptive, decision-making task distinguishes between social – implemented by a Trust Game – and non-social sources, as well as risk and ambiguity as underlying types of uncertainty. Our main result shows that individual beliefs regarding reciprocity likelihoods in both the Trust Game and the lottery influence the amount invested. Subsequently, only the investment amount in the Trust Game parametrically modulates anticipatory reward and outcome evaluation in the ventral striatum. This study demonstrates a first approach at using participants’ subjective sets of beliefs to examine reward processing. We discuss its potential promise, outline some limitations, and propose follow-up studies to extend the current approach.

## Introduction

Decision-making under conditions of uncertainty, that is, when we do not know the exact future outcome of our choices, is generally guided by beliefs we have about the world at large (Savage, 1954). These beliefs typically act as subjective probabilities, derived from a combination of specific prior knowledge, received information, and expertise in a particular domain (Fox and Tversky, 1995; Fox and Weber, 2002). For example, imagine you want to invest part of your capital in a mutual fund. There are thousands of options, but after reading many sources of information you select a few mutual funds. You will only learn if your assessment of these selected mutual funds was satisfactory after evaluating their quarterly holdings. While this is a difficult task in itself, the choice process can be even more complicated when decisions are made in direct interaction with another person, for example when we are deciding to trust or reciprocate with other people. To extend our previous example, imagine, as an alternative to investing in mutual funds, you opt to invest part of your capital in some startup businesses. You carefully decide which proposals to select based on your expectations about which entrepreneurs will successfully execute their business plan. However, you will only learn if your expectations were correct when, after several months or years, you receive the financial statements of the companies you funded. In particular, you are interested in reviewing the projects you expected to do well and for which you anticipated a high return on investment.

Decision-makers only ever get a full picture of the relationship between their beliefs and decisions by examining the eventual outcomes of these choices, which then offer the opportunity to learn whether their initial expectations were met, or whether they had in fact incorrectly assessed them. Prior to learning the actual outcomes however, one can imagine that anticipated rewards might increase in correspondence to an individual’s beliefs, i.e., the higher the beliefs of obtaining a reward in the future, the higher the anticipation of reward.

Though many studies have investigated the neural underpinnings of reward anticipation and outcome delivery with tasks such as the monetary incentive delay task (MID; Knutson et al., 2000) or the card guessing task (Delgado et al., 2000) in a lottery context, or a repeated Trust Game in a social context, to the best of our knowledge there has been no exploration to date of using decision-makers’ own, subjective, beliefs about the evaluation and subsequent outcome of their choices. Therefore, in this study we investigate reward anticipation and outcome when the reward “cue” is a function of prior internal evaluations as opposed to the standard method of using an externally provided cue association.

When we refer to beliefs, specifically we mean participants’ inherent priors, which are not manipulated in any way in this study, but which have formed based on previous personal, likely idiosyncratic, experiences. Our procedure is therefore different from studies which explicitly let participants form priors based on some experimental interaction, for instance a ball-tossing game with fictional players and specific behaviors (Fareri et al., 2012) or vivid descriptions of partners’ life events as to establish moral characters (Delgado et al., 2005). Furthermore, our study does not investigate social learning *per se*, as here the decision-making and outcome phase are separated in time (Fareri et al., 2012, 2015). After the outcome phase, participants review their prior decisions, but, importantly, do so without the possibility to change these previous choices. This has the effect of minimizing learning processes that may take place during the experimental process, as this is not the key feature of our study.

In tasks like the aforementioned MID and card guessing task, the decision-maker must perform a certain action correctly – a rapid reaction time in the MID and a correct guess in the card task – in order to receive a monetary reward. The essential feature of these games is that, before the required action, players learn that certain visual cues are associated with specific gains or losses, indicating either how large the monetary reward is or how much they can avoid losing if they perform the required task successfully. In the social domain, similar cues can be provided to denote a good or bad social partner. For instance, in Fouragnan et al. (2013), participants were told that triangles indicated game partners with low scores in a social orientation task, whereas circles indicated high social orientation scores.

Here, we are interested in naturally occurring individual beliefs, not induced by establishing specific cue-outcome relations. We examined these beliefs in the context of a decision-making task which distinguishes between both sources and types of uncertainty. We define sources here as uncertainty measured in social and non-social settings, which we operationalize with a Trust Game and a lottery mechanism, respectively.

In the Trust Game a sender invests a certain amount with a receiver based on beliefs she has regarding the receiver’s likely reciprocation, and therefore tries to reason about her partner’s trustworthiness. In the lottery context, the investor will analyze how much to invest with a random mechanistic device and is likely to use introspection, based on (any) experience with outcomes decided via such mechanisms, for example roulette or a coin toss. By using participants’ own belief sets it could be that participants rely more on these beliefs in a social context (Chang and Sanfey, 2011). That is, for example, correct beliefs regarding lottery outcomes are perceived as good luck, yet correct beliefs in a social situation are more likely perceived as a signal of personal success in properly assessing the social situation (Trautmann et al., 2008). Therefore, we are interested here in investigating whether social and non-social sources of uncertainty may influence belief-mediated anticipatory rewards in different ways.

In addition to exploring the relative *sources* of uncertainty, our study also distinguishes between *types* of uncertainty. By types of uncertainty, we refer to risk and ambiguity, which are events characterized by known objective probabilities and unknown probabilities, respectively (Wakker, 2010). A few studies have focused on the neural differences of anticipated rewards when cue-reward pairs are associated with either known probabilities (risk) or unknown probabilities (ambiguity). These studies show a distinct pattern of brain activation between anticipatory rewards under conditions of risk vs. ambiguity (Volz et al., 2003; Tobler et al., 2006), and are in line with primate studies which show that dopaminergic modulation of rewards varies across probability distributions (Fiorillo et al., 2003). By employing two types of uncertainty in this study, we can investigate both anticipated rewards that are a function of participants’ subjective beliefs (ambiguity), as well as objective probabilities we provide (risk).

In humans, the neural mechanisms of both the evaluation of reward decisions and their associated outcomes are mostly observed by bilateral activation of the ventral striatum (Bartra et al., 2013). This activity has been observed in a wide variety of outcome modalities. For example, activation in the ventral striatum, whose axons receive dopaminergic input from the ventral tegmental area (VTA) in the midbrain (Schultz, 1998), has been observed for monetary rewards (Knutson et al., 2001; Knutson and Greer, 2008), food (O’Doherty et al., 2002; Hare et al., 2008, 2009), social cooperation (Rilling et al., 2004; Davey et al., 2009; Jones et al., 2011; Korn et al., 2012; Lin et al., 2012; Powers et al., 2013) and even the punishment of others (Singer et al., 2006). Relatedly, in multiround trust games, Bellucci et al. (2017) found in a meta-analysis that the decision to trust also activated the ventral striatum, which they inferred to be likely associated with reward prediction error signals. However, during the feedback stage of this task, the dorsal striatum was active, which according to the authors was likely related to reinforcement learning processes.

In a similar manner to how anticipatory reward mechanisms operate when a previously learned cue is presented, we expect that people anticipate rewards when awaiting outcomes of decisions that were mediated by specific internal beliefs. When the investor in our earlier example anticipates a higher return from certain business projects, we would predict that these expectations would lead to increased reward anticipation prior to learning how these particular projects fared. Mechanistically, we hypothesize that this process is mediated by activation in the ventral striatum when participants are anticipating the potential outcome of their rewards. Though anticipating rewards in both social and non-social settings are thought to be processed in the striatum (Lin et al., 2012), our earlier hypothesis that participants might rely more on their beliefs in a social context could imply that we find stronger activation in the ventral striatum comparing between the Trust Game and a matched lottery task. With regard to types of uncertainty, there is evidence that predicting outcomes under various levels of uncertainty as compared to certainty activates the ventral striatum bilaterally (Volz et al., 2003). Therefore, with regard to ambiguity, we hypothesize greater ventral striatal activation for the anticipation of ambiguous as compared to risky outcomes. Lastly, during outcome delivery, we expect to observe activation in the ventral striatum as a function of the magnitude of the reward, that is, as a function of participant’s own earlier investment choices.

To examine this question experimentally, namely the neural mechanisms of belief-mediated anticipatory rewards and reward outcomes, an essential feature of our design is the careful elicitation of individual beliefs prior to decision-making. If we observe that participants’ decisions are indeed guided by their beliefs, we can then investigate the associated neural response as participants await and receive the respective outcomes. Importantly, this also optimally requires a clear and non-deceptive incentive scheme, as dopaminergic modulation is primarily observed when rewards are actually valuable in an uncertain environment (Schultz, 2010).

Taken together, this study aims to test how internally constructed beliefs, as opposed to objective cue-outcome associations, impact the neural mechanisms of reward anticipation and the subsequent delivery of rewards. Based on substantial pre-existing evidence that both reward anticipation and reward receipt are coded in the ventral striatum (Bartra et al., 2013), we hypothesize that both belief-mediated anticipatory rewards as well as reward receipt itself will activate the ventral striatum. We explore this question using a novel incentivized decision-making task that distinguishes between both types and sources of uncertainty.

## Materials and Methods

### Participants

A total of 26 participants (mean age = 22, 50% female) were recruited for this study via the online recruitment system SONA of the Donders Institute for Brain, Cognition and Behavior. Students with a psychology or economics background were excluded due to concerns about, respectively, suspicions regarding the veracity of the actual social interaction and a prior detailed understanding of game theoretic behavior.

Three of the 26 participants were excluded from our sample prior to analysis. One participant said that he did not believe the real human interaction and the incentive scheme after the experiment. Data for two participants were lost due to technical issues; the head coil was not applied correctly and the MRI data was not transferred appropriately. Furthermore, three participants were removed after analyzing all behavioral data as responses were very erratic, differed more than two standard deviations from mean responses and revealed clear misunderstandings (e.g., betting on scenarios with 0% chance to win) in one case and no variation in investment levels in the other two cases. Therefore, unless explicitly noted, analyses reported here are based on 20 participants (mean age = 22, 11 females and 9 males). Finally, this study was approved by the local ethical committee.

### Design and Procedures

The full experiment consisted of two parts, a decision phase and an outcome phase, separated by a short break. The focus of this manuscript is on the outcome phase. As the outcomes stem from the decision-making phase, we explain the setup below to be able to explain how outcomes were presented to participants.

On each trial, participants received an endowment of 10 tokens (which were later exchanged for cash). Participants could decide to invest any number of these tokens in either a human partner (social source) or a lottery (non-social source), depending on the experimental condition, with the investment amount then tripled by the experimenter. Additionally, there were two different types of uncertainty regarding the likelihood of their investment being repaid, that of risk and of ambiguity. This resulted in a total of four experimental conditions, explained in detail below.

In the *social* condition, we employed a standard Trust Game (Berg et al., 1995). The fMRI participant, termed the sender, had their (tripled) investment transferred to another player, known as the receiver. This receiver could then decide to either keep all this investment, or return half of it to the sender. If half was sent back, the sender was obviously better off than if they had transferred nothing, but at the time of decision faced uncertainty as to whether the receiver would reciprocate his or her trust. In the social context, participants placed an investment under two types of uncertainty: they explicitly knew the probability of being paired with a reciprocating receiver, known as the risky trust game (RTG), or they did not receive any probabilistic information regarding reciprocity, known as the ambiguous trust game (ATG).

Receivers’ choices were collected during a behavioral session prior to the fMRI experiment. Receivers made a binary choice to either return or keep the investment should a positive investment be received from the sender. Receivers could not condition their choice on the different investment amounts the sender could potentially invest with the receiver. Thereby our fMRI participants, in their role as sender, only acted upon beliefs regarding receivers’ trustworthy behavior, and their decisions were not confounded by other potential motives, for example signaling trust to receivers (McCabe et al., 2003) or eliciting positive reciprocity (Houser et al., 2010).

In the *non-social* condition, participants’ outcomes were resolved via a typical Ellsberg lottery design (Ellsberg, 1961). They bet on the color of a marble drawn from an urn, with this marble either a “winning” or “losing” color. Again, the fMRI participant decided on a transfer, receiving back either half of the tripled investment (if a winning colored marble was drawn), or alternatively losing their entire investment (if a losing colored marble was drawn). In this condition participants also faced two types of uncertainty. In the risky lottery (RLOT), participants knew the probabilities of drawing a marble with a winning color, whereas in the ambiguous lottery (ALOT) this probability was unknown.

We created risky and ambiguous trials in both social and non-social contexts by introducing a group principle to the general feature of the games discussed above. In the Trust Game, we grouped nine decisions made by nine different receivers. One receiver was randomly drawn from the pool of nine and matched to the MRI participant’s investment choice. In the lottery, there were nine marbles in the urn. One randomly drawn marble from this urn determined if the participant received half of his tripled investment.

**FIGURE 1:**
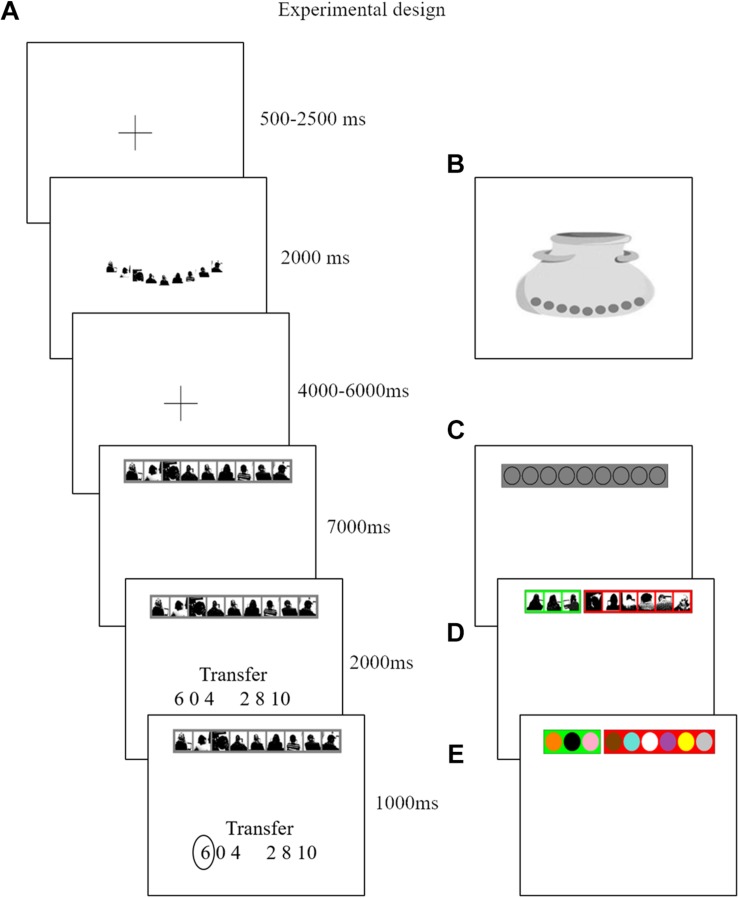
Each trial consists of six screens. Panel **(A)** is an example of a trial from the ATG. The second screen indicates the source of uncertainty. Nine silhouettes are displayed when participants are in a social context. Nine marbles are displayed when participants face a lottery context Panel **(B)**. The fourth screen is the decision screen. They are instructed to decide how much to transfer here. As the six possible transfer options appear in a random order on the next screen, they are unable to prepare for a specific button press. On the last screen we confirm their choice. In the ATG Panel **(A)** nine silhouettes on a gray background indicate that no information is given about the distribution of receivers that decided to send back half or keep the investment. To illustrate the tailor-made structure of our design, we assume a participant who believes three out of nine receivers will reciprocate. In the ALOT Panel **(C)** the participant receives instruction that three out of nine colors that can be used in any combination in this lottery are winning colors. In this way we align underlying subjective probabilities between the ATG and ALOT. In the risky trials we align individual’s beliefs to objective probabilities. A participant who believes three out of nine receivers will reciprocate, will most often face a RTG, which is composed of three receivers (green background) that decided to send back half of any received investment versus six receivers (red background) that decided to keep their investment Panel **(D)**. Finally, in the RLOT the urn is composed of all nine colors out of which three are winning colors (green background) and six are losing colors (red background) Panel **(E)**.

In the social context participants have underlying prior beliefs about the reciprocal behavior of receivers in general, and receive the following information as part of the instructions. We provided basic information regarding the pool of trustees, e.g., age, gender, study, hobbies – which were answered by the trustees after they had placed their reciprocating decision. Any difference fMRI participants, in their role as trustor, reveal about trustees’ reciprocating behavior is based on the same information all of them received and is therefore likely the result of differences in reciprocating behavior in general. Therefore it is important that we control for these beliefs in order to rule out inconsistencies in these underlying likelihoods and objective probabilities across our four experimental settings. For instance, imagine a sender who thinks that five out of nine receivers are likely to reciprocate. If this participant is confronted with a RTG where six out of nine receivers decided to transfer back half of the investment, we cannot assess whether differences in investment behaviors between both scenarios are caused by the type of uncertainty, or by a mismatch between subjective probability of 5/9 in the ATG and the objective probability of 6/9 in the RTG. Therefore, we elicited individual beliefs in the ATG before participants made decisions in our experimental setting. With an incentive-compatible belief elicitation technique (quadratic scoring rule, e.g., see Schlag et al., 2015), we asked how many receivers out of the pool of nine they thought would reciprocate their investment. This belief is then used to present participants with *belief-corresponding* scenarios in the experimental settings. Essentially, individual beliefs entailed a tailor-made trial structure for each participant (see Figure 1 for an overview and example of the experimental setup). By implementing this feature, we made sure that beliefs are aligned in our four settings. This enabled us to investigate expected reward signals by examining the effect of both source and type of uncertainty, taking into account participants’ naturally occurring beliefs.

**FIGURE 2:**
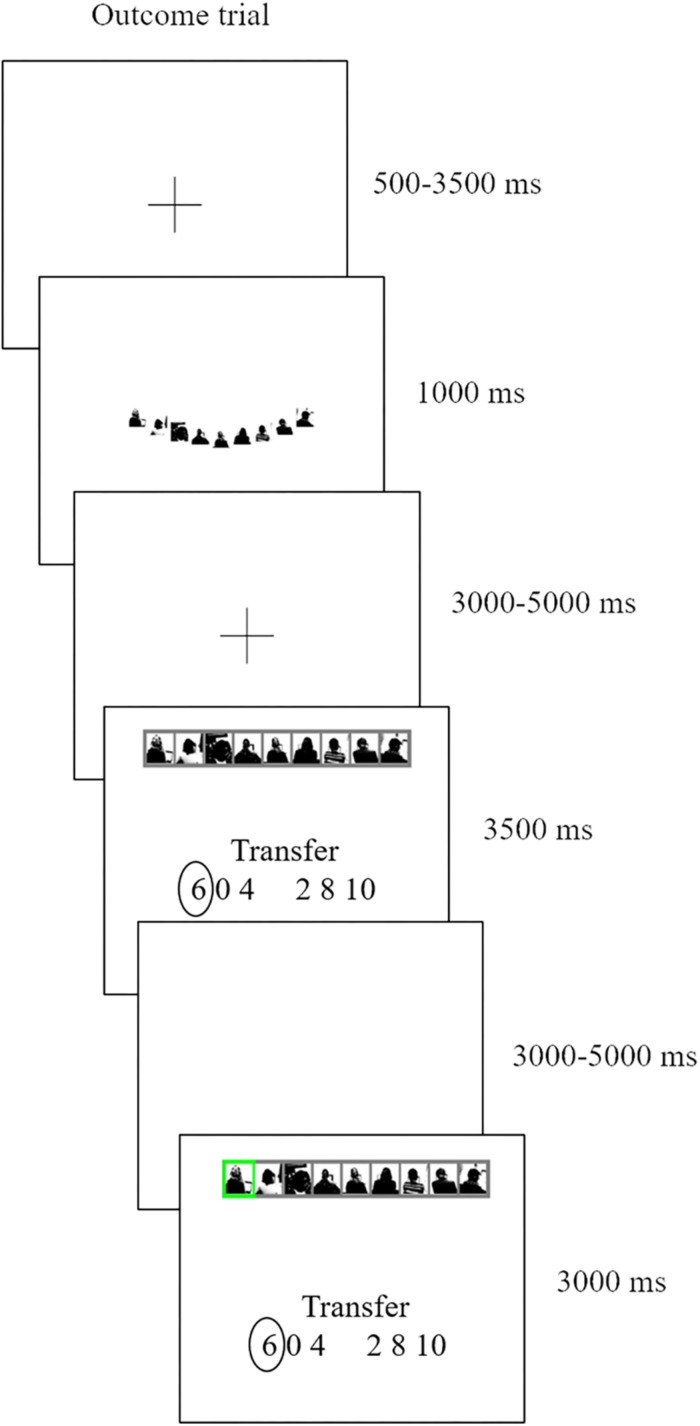
An outcome of an ATG trial. We took pictures of receivers while they were seated behind a laptop. The pictures only show receivers’ silhouettes in black and white and no facial features are shown.

To reiterate, we focus here solely on the outcome phase, that is, the revealing of decision (either trust or lottery) outcomes after all decisions have been made. Participants passively reviewed their previously made choices and then saw the respective outcome (see Figure 2 for a trial). During this outcome phase our primary focus is on the 3500 ms time period when participants are reminded of their earlier investment choice, and then await the outcome. We term this moment the *anticipation screen.* They then see the actual outcome of that trial, when a randomly selected receiver (social condition) or marble (non-social condition) is selected (final screen Figure 2, henceforth referred to as the *outcome screen)*. A selected receiver or marble highlighted in green indicates a winning trial, and when colored red indicates a losing trial.

Receivers’ decisions were collected during behavioral sessions, which took place at the Nijmegen School of Management decision laboratory. The fMRI experiment took place at the Centre for Cognitive Neuroimaging at the Donders Institute for Brain, Cognition and Behavior. The fMRI task was presented using Matlab Psychtoolbox (Kleiner et al., 2007). Participants read instructions and performed a belief elicitation task as part of the instructions (75 mins in total) before they were placed in the MRI scanner for approximately 60 min. The fMRI experiment consisted of the decision-making phase and outcome phase. After the decision-making phase, they saw a total of 88 outcome trials in the scanner, equally divided between trust and lottery outcomes (during the decision-making phase, participants also made choices when the chance of reciprocation was 0%, respectively, 100% chance. We excluded these decisions during the outcome phase as there is no uncertainty and thus no influence of individuals’ beliefs regarding their outcome). There were 15 outcome trials for each experimental condition and in addition there were filler trials for other probabilities in the RTG and RLOT that did not match participants’ beliefs. This provided greater variety in decision contexts, and also made it more difficult for participants to assess the individually tailor-made structure.

The outcomes were presented in 18 blocks, with each block consisting of five trials of either trust or lottery outcomes (four outcome trials for block 17 and 18). Within each block, both risky and ambiguous trials were presented in a random order. To enhance attention to the outcome phase, we introduced payment screens. After every two blocks, two outcomes were randomly selected, one from the lottery and one from the trust condition, which counted toward participants’ earnings. Each token was converted to 10 eurocents.

After the experiment subjects were paid out in cash dependent on their choices and randomly selected outcomes, and the accuracy of their stated beliefs. Notably, no deception was used in this experiment. Please see the appendix for the instructions and a detailed explanation of the payment scheme.

### Image Acquisition and Preprocessing

Functional neuroimaging data was collected on a 3-Tesla Siemens MRI system (Skyra) at the Donders Centre for Cognitive Neuroimaging in Nijmegen, Netherlands. Images were acquired using a 32-channel head coil, with a standard multi-echo imaging pulse T2^*^-weighted sequence (field of view = 224 mm, matrix = 64 × 64, repetition time (TR) = 2390 ms; echo times (TE) = 9.4, 20.6, 32.0, 43.0, and 54.0 ms, flip angle = 90°, slice gap = 0.5 mm). Using a multi-echo sequence provides a better signal-to-noise ratio for brain areas susceptible to dropout, while allowing for scanning of the whole brain (Poser et al., 2006). One whole-brain volume consisted of thirty-one ascending slices (slice thickness = 3.0 mm, voxel size = 3.5 mm × 3.5 mm × 3.0 mm). For each participant we acquired a high-resolution anatomical T1-weighted image (MPRAGE; 192 slices; TR = 2300 ms, voxel size = 1 mm × 1 mm × 1 mm). Participants’ heads were loosely taped to the coil within the scanner in order to limit movement during image acquisition.

fMRI data analysis was performed using SPM12 (Statistical Parametric Mapping; Friston et al., 2007). Prior to preprocessing we combined and realigned the five read-outs acquired via the multi-echo sequence by using standard procedures described by Poser et al. (2006). The first five volumes, acquired prior to task initiation, were used to estimate the weighted echo time per voxel for optimal echo combination including allowing T1 equilibration effects. These five volumes were then discarded from the analysis (Poser et al., 2006). After echos were combined, preprocessing consisted of slice-timing to the middle slice, co-registration of the functional images to the anatomical images, segmentation of the functional and anatomical image, and normalization to the Montreal Neurological Institute (MNI) template using the segmentation parameters. Functional images were then smoothed with a Gaussian kernel of 8 mm full-width at half maximum (FWHM).

### Data Analyses

#### Behavioral Parameters

In this study we were interested in the question of whether decision-makers’ beliefs about the outcomes of their choices would act as a cue for reward anticipation, and whether this might differ across conditions. In the RTG and RLOT participants do not face uncertainty as they receive objective probabilities (in line with the beliefs we elicit in the ATG), which naturally act as cue for reward anticipation.

In the ambiguous social context (the ATG), we elicited beliefs regarding the reciprocity of receivers before participants made investment decisions during the experiment. During the decision-making phase of this experiment, we observe participants’ actual investment choices and assume they stem from their individual subjective beliefs. It is therefore crucial that we establish a relationship between participants’ *a priori* beliefs and the investment choices they make in the ATG and the ALOT. Therefore, we will first examine whether indeed participants base their investment choices in the ATG and ALOT on their subjective expectations, and subsequently test whether participants’ investment levels different across our experimental conditions. These analyses consist of a linear mixed effects model (estimated with the toolboxes lme4 and lmerTest in R). The results section details the variables, random intercepts, and slopes included in this model.

#### Neuroimaging Analyses

To study the neural mechanisms of reward anticipation and outcome delivery, the primary explanatory variables (EV) of our general linear model (GLM) examined the BOLD response during trials in which participants reviewed their previously made choices and awaited their outcome (fourth screen in Figure 2). Four EV’s indicated the onset of the anticipation screens, modeled for a duration of 3500 ms, when participants reviewed decisions from the RTG (belief-corresponding risky trials), ATG, RLOT (belief- corresponding risky trials), and ALOT. To examine whether participant’s investment behavior served as a cue that would trigger expected rewards, we added this variable as parametric modulator to these four EV’s.

Other EV’s in this model included the other review decisions from the RTG and RLOT filler trials (not corresponding to participants’ beliefs), the trust or lottery cue (second screen in Figure 2), trials in which participants had not made a choice within the required 2 s (modeled at the onset of the anticipation screen for the full duration of the remainder of the trial), one outcome screen that coded a “win” (investment gets transferred back), one outcome screen that coded a ‘loss’ (participant loses investment), and finally one EV that modeled the nine payment information screens. The remaining events are the fixation and blank screen, which are therefore considered the implicit baseline.

When we were specifically interested in analyzing the BOLD responses of the actual outcomes, separated as wins and losses, we added the investment choices as parametric modulators to the outcome period, and entered these as the first variables to our model, otherwise similar as the model discussed above, in order to allow for sufficient explanatory variance regarding these parametric modulators.

All regressors were modeled with a canonical hemodynamic response function. To account for motion, we included the six head movement parameters together with their squared value and the temporal derivatives as nuisance regressors. A standard high-pass filter (cut-off 128 s) and auroregressive AR (1) model were used during the GLM analysis to account for possible slow-frequency drifts and temporal autocorrelation, respectively.

Our primary contrasts of interest are the anticipated rewards, as a function of the earlier chosen investment levels, re-evaluated during anticipation compared to implicit baseline, the specific neural mechanisms of anticipating outcomes as a function of source (social: anticipation ATG and RTG as compared to non-social: anticipation ALOT and RLOT), and comparing types of uncertainty (risk: RTG and RLOT vs. ambiguity: ATG and ALOT). Furthermore, we examine the amount won (lost) during the outcome phase, indicated by the investment level being reciprocated (held), compared to implicit baseline.

For the specified contrasts outlined above, one-sample *t*-tests were performed as second-level models to analyze group effects. Participants’ beliefs were added as a covariate at the group level. Statistical maps with an initial threshold of uncorrected *p* < 0.001 were established and were subsequently corrected for multiple comparisons using a Family Wise Error corrected cluster threshold of *p* < 0.05. As our hypotheses are centered on the role of the striatum during belief-mediated anticipation and outcome, we apply a small volume correction based on an *a priori* region defined by meta-analysis (Bartra et al., 2013), using specific coordinates for left striatum [−12, 12, −6] and right striatum [12, 10, −6], each with a radius of 10 mm.

Finally, the raw data and code used here will be made available by the authors to any qualified researcher.

## Results

### Beliefs and Decision-Making Under Ambiguity

Individual beliefs regarding the likelihood that receivers will reciprocate varied substantially. Some participants indicated quite low belief in receiver reciprocity, expecting only two or three of nine receivers to reciprocate their investment. On the other hand, some participants believed that six of nine receivers would return their investment.

**FIGURE 3:**
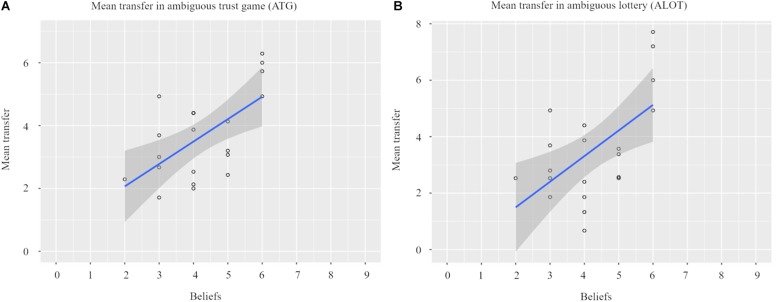
Elicited beliefs regarding receivers’ reciprocity influenced chosen transfer in the ambiguous trust game Panel **(A)**. Based on individual beliefs, participants received a matching amount of winning colors in the ambiguous lottery. Participants used this information, given during instructions prior to the experiment in the MRI scanner, as transfer positively increased as a function of the amount of winning colors Panel **(B)**.

Figure 3A illustrates that individual beliefs, elicited prior to the investment choice, positively correlated with the amount they subsequently invested in the ATG (*Pearson’s r* = 0.620, *p* = 0.004). That is, the larger the number of reciprocators that our participants thought would be present in a group of nine receivers, the more tokens they were willing to invest.

We also found a positive relationship between the amount of winning colors and participants’ investment choices in the ALOT (*Pearson’s r* = 0.587, *p* = 0.006, see Figure 3B). Thus, as expected, in both social and non-social contexts, the higher the subjective probability of receiving half of the tripled investment back, the more tokens participants were prepared to invest.

Although these results may appear intuitive, they are important for the neuroimaging analyses. When we add participant’s investment choices to our fMRI models we can reliably state that these investments are guided by their individual beliefs. Any difference we find across conditions is therefore unlikely to be the result of a mismatch between subjective probabilities (based on participants’ beliefs from the ATG), the underlying likelihood in the ALOT, or objective probabilities in the risk treatments.

**FIGURE 4:**
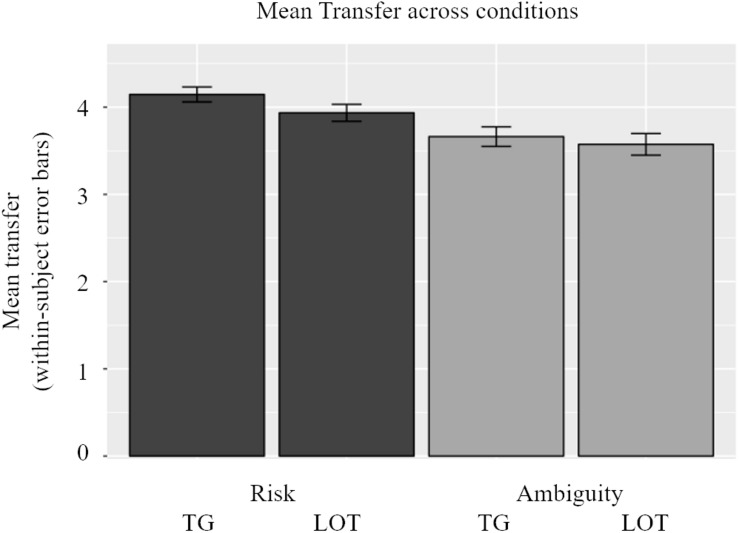
Overview of transfer choices across conditions. Participants invest less in the ambiguous conditions [both in Trust Game (TG) and lottery (LOT)] than the risky conditions. This effect is highly influenced by beliefs. The general pattern of ambiguity aversion only holds for participants with low beliefs in trustees (and number of winning colors). There was no effect of sources of uncertainty: participants do not alter their transfer between the TG and the LOT.

Participants’ beliefs also interacted with our experimental conditions resulting in interesting investment patterns in the Trust Game and lotteries. In a companion paper we focus exclusively on the decision-making phase and present its neuroimaging analyses – here we only look at the outcome phase in relation to beliefs – but for clarity we provide a short behavioral overview of investment behavior here. The mean transfer in the experiment, across conditions and subjects, was 3.83 tokens. In Figure 4, participants’ transfers are shown across conditions. In general, participants invested more in the risky conditions than in the ambiguous conditions, illustrating ambiguity aversion. This general pattern, however, was strongly influenced by individual beliefs, namely that the higher were beliefs regarding reciprocity in the ATG (and number of winning colors in the ALOT), the more ambiguity averse behavior was displayed. This result is similar to findings from experimental economics, which show variability in ambiguity aversion along the probability distribution (Trautmann and van de Kuilen, 2014). These results are confirmed by a linear mixed-effects model which consisted of participants’ transfers as the dependent variable, and type (risk vs. ambiguity) and source (Trust Game vs. lottery) of uncertainty as independent factors, along with gender, participants’ beliefs, trial number, and an interaction of beliefs and both experimental factors. A random intercept and two random slopes accounted for clustering at the participant level and repeated trials within experimental conditions. Confirming the bivariate correlation between beliefs and investment choice, the mixed effects model underlined the significance of participants’ beliefs (β = 0.891, *p* = 0.002 via Satterthwaite’s method) and their interaction with the type of uncertainty (β = 0.538, *p* = 0.025 via Satterthwaite’s method). Although the variable trial number was also negatively significant (*p* = 0.027) – indicating that as participants progress through the experiment they transfer less – its economic significance was rather small (β = −0.006).

### Imaging Data

We first focused on the observed neural activity during the anticipation phase. To check whether participants were actually observing their previously made choices, we first examined the BOLD signal during all anticipation screens (fourth screen in Figure 2). Various brain regions were active – parahippocampal cortex (peak activation: −8, −52, 0, 1282 voxels, *p* < 0.001), dorsolateral prefrontal cortex (peak activation: −32, 10, 56, 63 voxels, *p* = 0.007), and control network (peak activation: −50, −52, 38, 64 voxels, *p* = 0.007) – which, based on the NeuroSynth database (Yarkoni et al., 2011), are very likely to be involved in attentional and memory processes.

**FIGURE 5:**
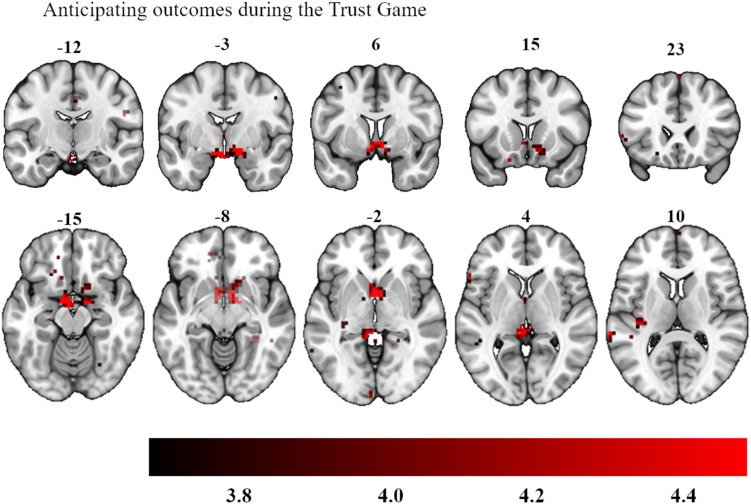
Bilateral activation in the ventral striatum as a function of participants’ investment choices during the anticipation of their social outcomes. The color map defines the strength of the contrast’s *T*-scores (Statistical maps with an initial threshold of uncorrected *p* < 0.001 were established and were subsequently corrected for multiple comparisons using a Family Wise Error corrected cluster threshold of *p* < 0.05).

More importantly, we then added participants’ investment level as parametric modulator, allowing us to ask whether trials on which the most tokens were invested showed a greater expected reward signal while participants reviewed their chosen investment prior to seeing the outcome. When we focused on investment choices during the anticipation phase across all experimental conditions (ALOT, RLOT, ATG, RTG), no subthreshold clusters were found. We then looked at the social and non-social anticipatory outcomes separately. Analyses here demonstrated that the more that was invested in the Trust Game, the more activation was observed bilaterally in the ventral striatum (peak activations: −4, 7, −7 and 6, 4, −7, 11 voxels, *p* = 0.025 after small volume correction, see Figure 5), whereas no regions surpassed this threshold during the lottery outcomes. A direct comparison of investment levels in the Trust Game versus the lottery also revealed an area in the ventral striatum bilaterally, as part of an area which extended into the orbitofrontal cortex (peak activations: −8, 21, −4 and −15, 35, −7, 18 voxels, *p* = 0.020 after small volume correction).

Next, we explored the different types of uncertainty anticipation, namely comparing risky versus ambiguous trials, but found no significant neural effects for this contrast. Even when we restricted the analysis to a functional ROI based on the contrast which described investment levels between Trust Game and lottery, we did not observe activation in this area.

**FIGURE 6:**
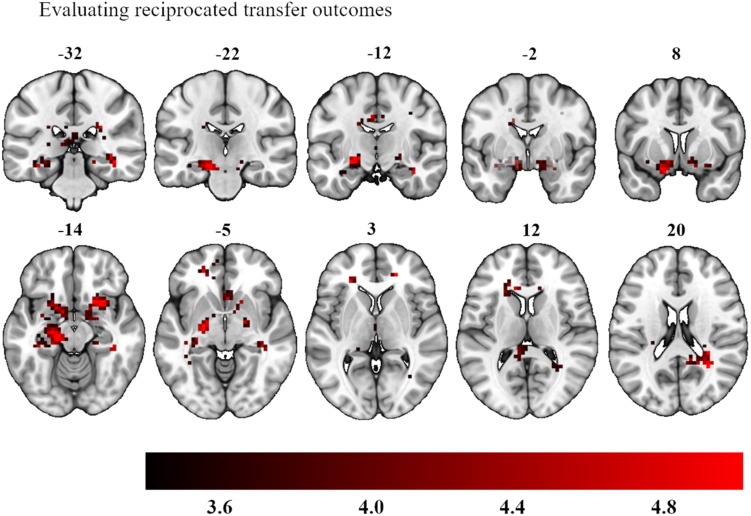
Bilateral activation in the amygdala, left ventral striatum and right dorsal striatum as a function of participants’ investment choices during the delivery of their outcomes. The color map defines the strength of the contrast’s *T*-scores (Statistical maps with an initial threshold of uncorrected *p* < 0.001 were established and were subsequently corrected for multiple comparisons using a Family Wise Error corrected cluster threshold of *p* < 0.05).

We also examined the question of neural differences when outcomes were finally resolved. We investigated the investment amount as a parametric modulator when experiencing a win during the outcome phase, collapsed across experimental conditions (last screen in Figure 2). This contrast yielded strong bilateral activation in an area encompassing the amygdala bilaterally, left ventral striatum and right dorsal striatum (left hemisphere peak activations: −22, −14, −10 and −18, 7, −18, 193 voxels, *p* < 0.001 whole brain analysis; right hemisphere peak activations: 20, −7, −7 and 20, 18, −10, 84 voxels, *p* = 0.003 whole brain analysis, see Figure 6). The investment amount as parametric modulator for a loss did not show any significant activation patterns.

We further investigated whether individual differences in attitudes toward social and ambiguity preferences might explain variance in neural data. Individuals’ social preferences were defined as a normalized score between −1 to 1 where a score above (below) 0 indicated a person who invested more (less) with a person in the TG than the lottery. Individuals’ ambiguity preferences were also defined as a normalized score between −1 to 1 where a score above (below) 0 indicated a person who was ambiguity averse (seeking). When we added social preferences as a covariate to the contrast which investigated neural differences in investment levels in the Trust Game versus the lottery, we observed the right motor and somatosensory cortex activation (*p* = 0.015 whole brain). Individuals’ ambiguity preferences as covariates for the contrast investment levels in the ambiguous versus the risky settings did not yield any significant neural findings.

## Discussion

Reward is an important and well-studied topic in the field of Neuroscience (Bartra et al., 2013). Initiated by innovative primate studies, a growing literature has emerged examining the putative dopaminergic modulation of reward (Schultz et al., 1997; Schultz, 1998). Our study sought to address scenarios when anticipated rewards stem from individuals’ own beliefs and subsequent decision-making, instead of relying on cue-outcome associations that are typically evident in tasks such as the MID and multiround Trust Games. In this experiment, we examined the strength of individual beliefs, their relationship with subsequent decisions, and their associated neural mechanisms when anticipating their outcomes. These questions were explored in a real-life decision-making context, in which outcomes were clearly (and non-deceptively) resolved. We asked whether these belief-mediated anticipated rewards were neurally processed in the manner of an expected reward signal, similar to how rewards are evoked through abstract cue-outcome associations.

Our decision-making task distinguished between social (Trust Game context) and non-social (lottery context) sources of uncertainty, as well as risk and ambiguity as types of uncertainty. Choices made by participants in both the Trust Game and the lottery tasks indicated clearly that underlying beliefs did in fact guide participants’ decision-making. Participants invested more when they expected a greater number of their potential game partners to reciprocate their investment in the ATG. Similarly, participants in the ALOT invested most when they knew a greater number of colors out of the nine possible colors would lead to a return on their investment. Subsequently, individuals’ investment behavior is also influenced by their beliefs: the higher beliefs regarding reciprocity were in the ATG (and number of winning colors in the ALOT), the more ambiguity averse behavior was displayed.

Our neuroimaging analyses then focused on whether these belief-related expectation signals were evident in brain regions related to standard cue-based reward anticipation. We found confirmatory evidence of this in the Trust Game. The greater the expectation of receiving a back-transfer in the Trust Game, the greater the investment amount that was made, and in turn the greater the activation in bilateral ventral striatum prior to the outcome being presented, as compared to anticipation in the lottery context. Anticipating the outcome of whether your investment is reciprocated by another person versus a lottery is likely more salient as it depends on subjective assessments of trusting and engaging with another people and its outcome results from their intentional behavior, which aspects are of course absent when interacting with a mechanistic device. Also, one consequence of our experimental approach is that participants in the ALOT did not actively have to form a prior belief. A feature of dopaminergic modulation of reward is that the more uncertain a reward is, the more information the consequent outcome will allow for updating of priors (Schultz, 2010). Although a different ambiguous urn was constructed on every trial in the ALOT, participants knew how many colors were winning colors. This feature might have reduced the uncertainty in the ALOT as compared to the ATG.

Our novel result illustrates that one’s own investment choice, modulated by one’s expectations regarding receivers’ reciprocating behavior, can serve as an anticipatory cue. Here though, the cue was neither externally created by character vignettes (Delgado et al., 2005) nor learned in a Pavlovian manner by pairing shapes to more or less trustworthy persons in a social context (Fouragnan et al., 2013), but was rather internally generated via participants’ own beliefs about the world. This finding illustrates that eliciting participants’ beliefs can be just as powerful in evoking anticipated reward signals as specifically pairing abstract cues with explicit (social) gains and losses.

We also showed that when participants were informed about a positive outcome – that their trust decision was reciprocated by a receiver in the Trust Game or that their marble was drawn in the lottery – the degree of their chosen investment level modulated the reward signal in the left ventral striatum and right dorsal striatum. These effects also highlight the potential of using participants’ own beliefs in a real-life decision-making task when examining reward and subjective value. Some other effects are also worth exploring further.

A well-established finding is that losses are coded in the ventral striatum (Bartra et al., 2013). However, experiencing a loss in this study, that is, when the amount invested was not returned, did not activate similar brain regions as compared to when a trial was “won.” Notably though, participants in this task did not actually lose money, but rather they lost the opportunity of winning more money by receiving a part of the tripled investment. When they lost, they still retained the non-invested number of tokens, thus perhaps minimizing the effect of the virtual loss. Moreover, it is found that positive effects are more likely to be coded in the striatum than negative effects (Bartra et al., 2013). These factors might explain this null finding with regard to experiencing losses.

Secondly, we also did not find neural differences in the anticipation of outcomes between ambiguous and risky contexts. Our experimental design differs from earlier explorations showing that various levels of uncertainty modulate expected reward in the ventral striatum (Fiorillo et al., 2003; Volz et al., 2003; Tobler et al., 2006). Namely, following standard practices in Economics (Wakker, 2010), here we clearly distinguish between risk and ambiguity, instead of varying uncertainty along a continuous distribution. Although decision-making under risk and ambiguity appear to be processed independently (Hsu et al., 2005; Huettel et al., 2006), anticipating their respective outcomes does not appear to differentially modulate neural processes. It might be that passively observing prior decisions does not sufficiently highlight the distinction between the types of uncertainty. Whereas revealing outcomes of social vs. non-social contexts emphasizes the role of the receiver and his intentions as compared to a non-intentional random mechanistic device, separating outcomes by types of uncertainty is likely not as compelling.

In a broader context, this is also a limitation of our experimental setup. We purposely separated the decision-making phase from the outcome phase, as we did not want participants to learn from the outcomes of their choices which could lead to potential belief adaptation across the experiment. While this means that our design can rule out learning effects, and that we can reliably use the beliefs elicited prior to decision-making, a downside of this procedure is that the re-evaluation of the choices that participants undertake is quite passive. Although we endeavored to enhance attention by including payment screens, we would ideally engage participants more intensively. Additionally it is worth noting that these results are based on a rather small sample size, and as such deserve follow-up exploration.

One interesting potential follow-up could be to design a dynamic experiment in which participants would be able to change future decision-making as a function of beliefs, which would presumably be updated as participants learned about the outcomes of prior choices, and beliefs could thus be elicited at various moments throughout the fMRI experiment. This would promote active engagement of both decision-making and outcome attention as well as the interaction between both phases as a function of belief updating, which moves experimental approaches closer to how trust and reciprocity are experienced in everyday life. This method could bridge two important directions in the field of Decision Neuroscience: namely, explorations of reward processing, which to date have rather neglected the role of participants’ inherent beliefs, and the analyses of beliefs, which have focused on how beliefs emerge and are shaped (Vilares and Kording, 2011) but have examined less the interaction with expected value processing. Our study offers a first attempt as to how participants’ own belief sets are employed in the reward processing in the context of trust and risky choice.

## Ethics Statement

This study was carried out in accordance with the recommendations of the local ethical committee with written informed consent from all subjects. The protocol was approved by the local ethical committee (CMO Arnhem-Nijmegen).

## Author Contributions

All authors contributed to the conception and design of the study, and revised, read, and approved the submitted version of the manuscript. KF carried out the statistical analysis and wrote the first draft of the manuscript.

## Conflict of Interest Statement

The authors declare that the research was conducted in the absence of any commercial or financial relationships that could be construed as a potential conflict of interest.
